# Epstein–Barr virus LMP1 induces focal adhesions and epithelial cell migration through effects on integrin-α5 and N-cadherin

**DOI:** 10.1038/oncsis.2015.31

**Published:** 2015-10-19

**Authors:** L R Wasil, K H Y Shair

**Affiliations:** 1 Cancer Virology Program, UPCI Hillman Cancer Center, Department of Microbiology and Molecular Genetics, University of Pittsburgh, Pittsburgh, PA, USA; 2 Lineberger Comprehensive Cancer Center, Department of Microbiology and Immunology, University of North Carolina at Chapel Hill, Chapel Hill, NC, USA

## Abstract

Epstein–Barr virus (EBV) is a γ-herpesvirus associated with human epithelial and B-cell malignancies. The EBV latent membrane protein (LMP) 1 is expressed in nasopharyngeal carcinoma (NPC) and promotes oncogenic intracellular signaling mechanisms. LMP1 also promotes a pro-migratory phenotype through potential effects on cell surface proteins, as expression of LMP1 induces an epithelial–mesenchymal transition (EMT) in epithelial cell lines. In this study, LMP1 was examined for potential effects on cadherin and integrin surface interactions, and assessed for biological effects on adhesion and motility to fibronectin. Expression of LMP1 in the non-tumorigenic epithelial cell line MCF10a induced an EMT-associated cadherin switch. The induced N-cadherin was ligated and localized to the cell surface as determined by triton-solubility and immunofluorescence assays. In addition, LMP1 induced the assembly of focal adhesions (FAs) with increased production of fibronectin in MCF10a and NP460hTERT-immortalized nasopharyngeal cells. Biochemical enrichment of fibronectin-associated proteins indicated that LMP1 selectively promoted the recruitment of integrin-α5 and Src family kinase proteins to FA complexes. Neutralizing antibodies to N-cadherin and integrin-α5, but not integrin-αV, blocked the adhesion and transwell motility of MCF10a cells to fibronectin induced by LMP1. LMP1-induced transwell motility was also decreased by Src inhibition with the PP2 kinase inhibitor and short hairpin RNAs. These studies reveal that LMP1 has multiple mechanisms to promote the adhesive and migratory properties of epithelial cells through induction of fibronectin and modulation of cell surface interactions involving integrin-α5 and N-cadherin, which may contribute to the metastatic potential of NPC.

## Introduction

Epstein–Barr virus (EBV) is a γ-herpesvirus associated with epithelial and B-cell malignancies. The majority of adults are asymptomatically infected, however, a subset develops cancer with increased risk upon immunosuppression.^1, 2^ EBV persists latently in B cells with sporadic reactivation, but can also infect oropharynx and gastric epithelia. The primary outcome of epithelial infection is lytic replication, but in persistent latent infection is associated with the oncogenic phenotypes of nasopharyngeal carcinoma (NPC) and gastric carcinoma.^3^ EBV-infected NPC represents approximately 78 000 new annual cancer cases, with higher incidence in specific geographical regions such as Southeast Asia.^1, 4^ Clonal EBV infection is consistently detected in NPC, and expresses a type II latency program that includes the expression of the membrane proteins latent membrane protein 1 (LMP1), LMP2A, LMP2B, the EBNA1 episome maintenance protein, and BART and EBER non-coding RNAs.^5^

The transforming and oncogenic potential of the putative viral oncoprotein LMP1 have been characterized both *in vitro* and *in vivo*. LMP1 transforms rodent fibroblasts, promotes growth in soft agar, forms tumors in nude mice and enhances the migration of epithelial cell lines.^6, 7, 8^ Transgenic mice expressing LMP1 from the epithelial-specific keratin-14 promoter have increased formation of hyperproliferative papillomas, and with LMP2A demonstrate co-operative induction of invasive carcinomas by chemical carcinogens.^9^ The immortalization of B cells by EBV is also dependent on LMP1 expression.^2^ LMP1 is a constitutively active transmembrane protein that interacts through the cytoplasmic domains (C-terminal activation regions—CTAR 1 and 2) with tumor necrosis receptor family adapter molecules (TRAFs) to activate oncogenic mechanisms including phosphoinositide 3-kinase (PI3K)/protein kinase B (Akt), epidermal growth factor receptor (EGFR), extracellular signal-regulated kinase (ERK) and nuclear factor kappa-light-chain-enhancer of activated B cells (NFκB).^6, 7, 10, 11, 12^ LMP1 may also regulate extracellular interactions through effects on junctional plakoglobin, E-cadherin and N-cadherin.^8, 13, 14, 15^ Plakoglobin and E-cadherin expression levels are decreased in NPC, but increased expression of N-cadherin correlates with advanced stage metastasis.^8, 16, 17^ Molecular characteristics of epithelial–mesenchymal transition (EMT) may contribute to metastasis, and one hallmark is the induction of a cadherin switch.^18^ In epithelial and NPC cell lines, LMP1 expression decreases junctional plakoglobin levels and induces a cadherin switch with EMT-associated morphological changes.^3, 8, 13, 14, 15, 19, 20, 21^ These molecular effects and increased actin polymerization contribute to LMP1-mediated epithelial cell motility and spreading, and suggest that LMP1 may modulate extracellular interactions involving integrin binding to the extracellular matrix (ECM) and cadherin ligations to neighboring cells.^8, 22, 23, 24^

There are few authenticated nasopharyngeal epithelial cell lines that are not immortalized by other viral oncoproteins or potentially contaminated with HeLa cells.^25^ In this study, the effects of LMP1 were largely assessed in the spontaneously immortalized non-tumorigenic, non-metastatic breast epithelial cell line MCF10a. In these cells, LMP1 induced features of EMT and increased adhesion and transwell migration to fibronectin. Inhibition of integrin-α5 or N-cadherin ligation, and Src kinase activity decreased LMP1-induced adhesion and motility. A novel induction of focal adhesions (FAs) by LMP1 was also identified in additional epithelial cell lines of nasopharyngeal (NP460hTERT) and non-nasopharyngeal (U2OS) origins. Further characterization by enrichment of FAs in MCF10a cells isolated with fibronectin-coated magnetic beads demonstrated increased levels of integrin-α5 and Src. These data indicate that LMP1 can modulate the recruitment of cell surface proteins resulting in the constitutive assembly of FAs and ligation of N-cadherin, providing mechanistic insight into LMP1-mediated induction of adhesion and motility to ECM in epithelial cells.

## Results

### LMP1 induces EMT markers in MCF10a cells

**Figure 1:**
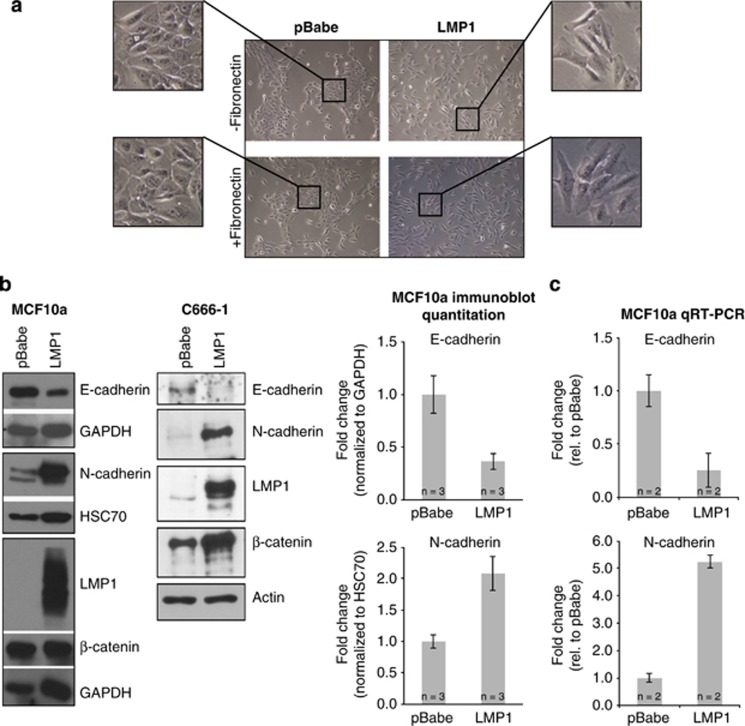
LMP1 induces EMT in MCF10a cells. (**a**) Comparison of MCF10a stable cell line morphologies seeded on fibronectin-coated and -uncoated surfaces by phase contrast. (**b**, **c**) Quantitation of E-cadherin and N-cadherin levels by immunoblot and quantitative RT–PCR analyses. Immunoblot comparisons were performed in MCF10a and C666-1 stable cell lines. Normalized fold-change is calculated relative to the pBabe control set to 1. '*n*' indicates the number of independent biological replicates and error bars indicate s.d. For quantitative RT–PCR, experiments were performed with technical triplicates and averaged from two biological replicas.

LMP1 induces a cadherin switch and promotes the migration of the NPC cell line C666-1.^8, 14^ To investigate the activity of LMP1 in a non-cancerous cell line, LMP1 was stably expressed by retroviral transduction in MCF10a cells, a cell line susceptible to EMT inducers such as transforming growth factor-β.^26^ In contrast to the epithelial morphology of pBabe vector control cells, LMP1 expression induced an ECM-independent elongated mesenchymal morphology on uncoated and fibronectin-coated surfaces (Figure 1a). LMP1 decreased E-cadherin protein levels approximately 70% and increased N-cadherin >2-fold in MCF10a cells, which was consistent in C666-1 cells (Figure 1b).^14^ However, β-catenin levels, a component of the cadherin junctional complex, were not consistently changed by LMP1 expression between cell lines (Figure 1b). N- and E-cadherin were modulated transcriptionally by LMP1 expression (Figure 1c). The change in cadherin expression and transition to a mesenchymal morphology indicate that LMP1 induces molecular and morphological features associated with EMT in MCF10a cells.

**Figure 2:**
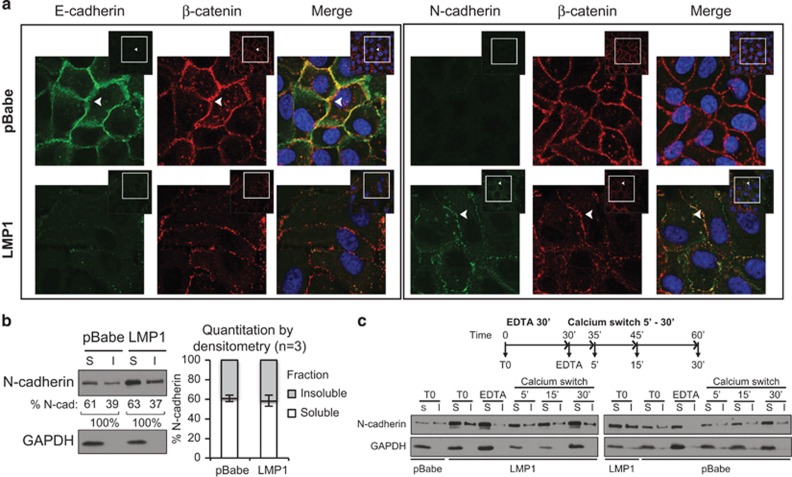
LMP1 induces a cadherin switch at the cell surface. (**a**) Immunofluorescence staining for E-cadherin, N-cadherin and β-catenin in MCF10a stable cell lines. Nuclei were stained with 4,6-diamidino-2-phenylindole (DAPI) and imaged by confocal microscopy. Inset, images were digitally zoomed to display staining at the cell periphery (arrows). (**b**) Triton-solubility and immunoblot analysis of soluble (S) and insoluble (I) N-cadherin from MCF10a stable cell lines. The percentage of N-cadherin in each fraction was quantified by densitometry from three independent experiments (*n*). (**c**) Ligation of cell surface N-cadherin in MCF10a cells was assessed by EDTA treatment followed by a calcium switch, and analyzed by immunoblotting for soluble (S) and insoluble (I) N-cadherin in a triton-solubility assay.

To evaluate potential changes in adherens junction-associated cadherins, confluent monolayers of MCF10a cells were analyzed for N-cadherin, E-cadherin and β-catenin (Figure 2a). In pBabe cells, N-cadherin was not detected, but E-cadherin and β-catenin colocalized at the cell periphery. In contrast, LMP1-expressing cells had weak E-cadherin staining with colocalized N-cadherin and β-catenin staining at the cell periphery, indicative of adherens junctions. N-cadherin C-terminal fragments may also regulate cAMP response element-binding protein (CREB)-mediated transcription, but cytoplasmic staining of N-cadherin was not detected indicating that LMP1-induced cadherin switch predominantly occurs at potential sites of intercellular interaction on the cell surface.^27^ To assess the levels of ligated N-cadherin, MCF10a cells were analyzed by a triton X-100-solubility assay. LMP1 increased soluble and insoluble N-cadherin, but the proportions of N-cadherin in each fraction did not change (Figure 2b). In comparison, the triton-soluble protein glyceraldehyde 3-phosphate dehydrogenase (GAPDH) was excluded from the triton-insoluble fraction supporting fraction purity (Figure 2b). To further investigate if ligated N-cadherin is increased by LMP1, MCF10a cells were analyzed by a calcium switch assay, in which N-cadherin ligation at the cell surface is disrupted by the chelating agent EDTA. The re-addition of calcium restores functional cadherins, which can be detected as a complex in the triton-insoluble fraction. Before treatment with EDTA (T0), insoluble N-cadherin was in both vector control and LMP1-expressing cells (Figure 2c). Treatment with EDTA for 30 min specifically decreased insoluble N-cadherin to trace levels, indicating effective disruption of ligated N-cadherin in both cell lines (Figure 2c). After EDTA removal and washes, less adherent cells may be recovered at early time points, which is reflected by the decrease in GAPDH levels at 5- to 15-min calcium switch. However, comparison of N-cadherin fractions from the same harvest indicates that ligation is rapidly re-initiated as early as 5 min in the triton-insoluble fraction (Figure 2c). These data support that the insoluble fraction contains ligated N-cadherin occurring at increased levels in LMP1-expressing cells; however, the amount of ligated N-cadherin was proportional to its relative abundance in each cell line. These data indicate that LMP1 induces EMT and increases N-cadherin expression and ligation at the cell surface.

### LMP1-induced migration to fibronectin requires N-cadherin and integrin-α5

**Figure 3:**
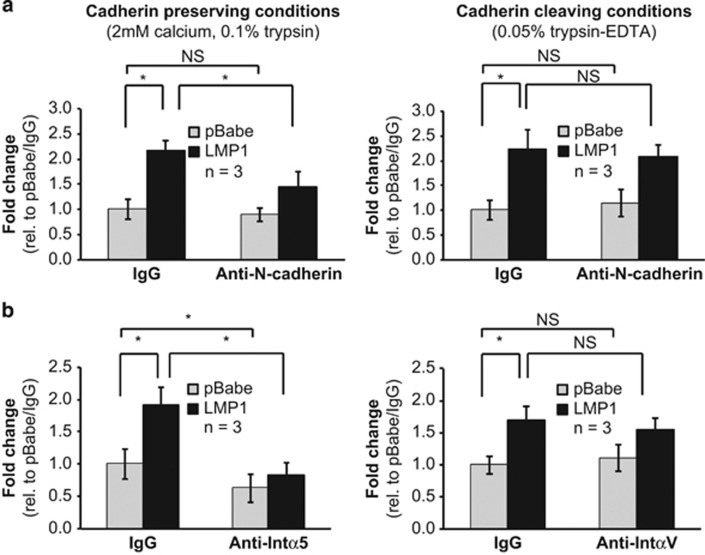
LMP1-induced migration of MCF10a cells to fibronectin is dependent on integrin-α5 and N-cadherin. MCF10a stable cell lines were assayed by transwell migration to fibronectin. Neutralizing antibodies to (**a**) N-cadherin, and (**b**) integrin-α5 and -αV, were assessed for effects on transwell motility. N-cadherin blocking was compared in cadherin preserving and cleaving conditions. Fold-change in migration was calculated from three independent experiments (*n*) with error bars indicating s.d., and compared by the Student's *t*-test. *P=0.01; NS, not significant.

LMP1 can enhance transwell migration of NPC cells to fibronectin, which may be mediated by enhanced intercellular and ECM interactions.^8^ In addition, loss of E-cadherin and plakoglobin contributes to LMP1-induced motility in multiple epithelial cell lines.^3, 8, 14, 15, 21^ To determine if LMP1 enhances MCF10a migration with the potential contribution of N-cadherin and integrins, transwell migration to fibronectin was assessed in the presence of neutralizing antibodies to N-cadherin, integrin-α5 or -αV. N-cadherin neutralization was evaluated in cadherin preserving and cleaving conditions. In cadherin preserving conditions, cells were dissociated by trypsin in the presence of calcium to protect cadherins from protease digestion, and filtered through a cell strainer to generate single cell suspensions.^28^ Expression of LMP1 significantly increased migration of MCF10a cells to fibronectin and treatment with N-cadherin neutralizing antibody decreased LMP1-induced migration (Figures 3a, *P*=0.01). In contrast, LMP1-induced migration did not decrease in cadherin cleaving conditions (0.05% trypsin-EDTA) with N-cadherin neutralizing antibodies (Figure 3a). These findings indicate that the blocking effect requires intact N-cadherin at the time of neutralization, and suggests that restoration of LMP1-induced migration post-cadherin cleavage may result from re-expression of surface cadherins, which could be restored within hours of trypsinization.^29^

Integrin-α5 and -αV are the principal α-integrins that bind fibronectin. To examine effects independent of cadherin interactions, transwell migration was assessed in cadherin cleaving conditions with neutralizing antibodies to integrin-α5 and -αV. Neutralizing integrin-α5 decreased migration of pBabe cells and reduced migration of LMP1-expressing cells to pBabe levels, supporting that integrin-α5 is a primary integrin for binding to fibronectin (Figures 3b, *P*=0.01). In contrast, integrin-αV neutralizing antibody, which inhibits LMP2A-induced migration to fibronectin, did not affect migration of pBabe or LMP1-expressing cells (Figure 3b).^30^ These results indicate that integrin-α5, but not integrin-αV, is required for LMP1-induced migration to fibronectin in MCF10a cells.

**Figure 4:**
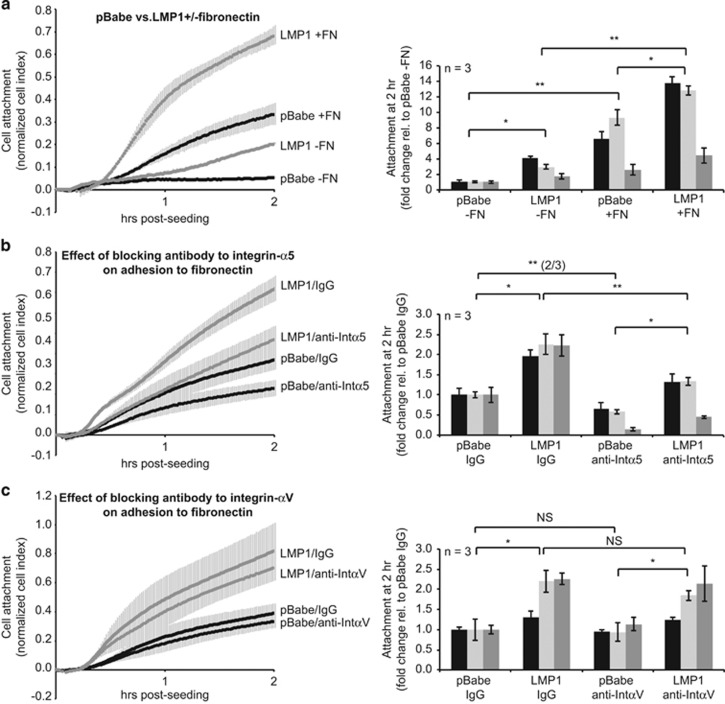
LMP1-induced adhesion of MCF10a cells to fibronectin is dependent on integrin-α5. Attachment of MCF10a stable cell lines was assayed by RTCA for 2 h post-seeding. (**a**) Comparison of MCF10a stable cell lines adhering to fibronectin (FN)-coated and -uncoated surfaces. (**b,** **c**) The effect of neutralizing antibodies to (**b**) integrin-α5 and (**c**) integrin-αV on the adhesion of MCF10a stable cell lines to fibronectin-coated surfaces. Normalized cell index values were calculated from electrical impedance readings as a result of cell attachment, and normalized to the starting value at the time of seeding. A representative cell attachment curve is shown, averaged from a min. of triplicate wells with s.d. displayed. At 2 h post-seeding, fold-change in attachment from three independent experiments (*n*) was calculated and analyzed by the Student's *t*-test. *P=0.01; **P=0.05; NS, not significant. In one comparison, the *t*-test was not significant in all three experiments and the number of experiments in which the *t*-test was significant is indicated in parenthesis (2/3, two out of three experiments).

To evaluate effects of LMP1 on ECM-mediated adhesion, cell attachment was continuously monitored for 2 h using the xCelligence RTCA instrument, which measures electrical impedance as a determinant of cell attachment. Attachment of MCF10a cells was compared on fibronectin-coated and -uncoated surfaces. Fibronectin increased attachment of both pBabe and LMP1-expressing cells (Figures 4a, *P*=0.05), confirming that MCF10a cells respond to fibronectin. LMP1 also increased attachment of MCF10a cells to uncoated surfaces (Figures 4a, *P*=0.01). Although the fold-changes in attachment varied between experiments, the increase was consistent across independent experiments (Figures 4a, 3/3 experiments). Neutralizing antibodies to integrin-α5 decreased attachment of pBabe cells (2/3, *P*=0.05), and consistently inhibited attachment of LMP1-expressing cells (3/3, *P*=0.05) to levels comparable to pBabe cells (Figure 4b). In agreement with the findings in transwell migration (Figure 3b), neutralizing integrin-αV did not affect attachment of pBabe or LMP1-expressing cells (Figure 4c).^30^ These data indicate that LMP1 enhances attachment of MCF10a cells to fibronectin through effects on integrin-α5.

### Induction of FAs by LMP1 is dependent on Src and contributes to enhanced migration

**Figure 5:**
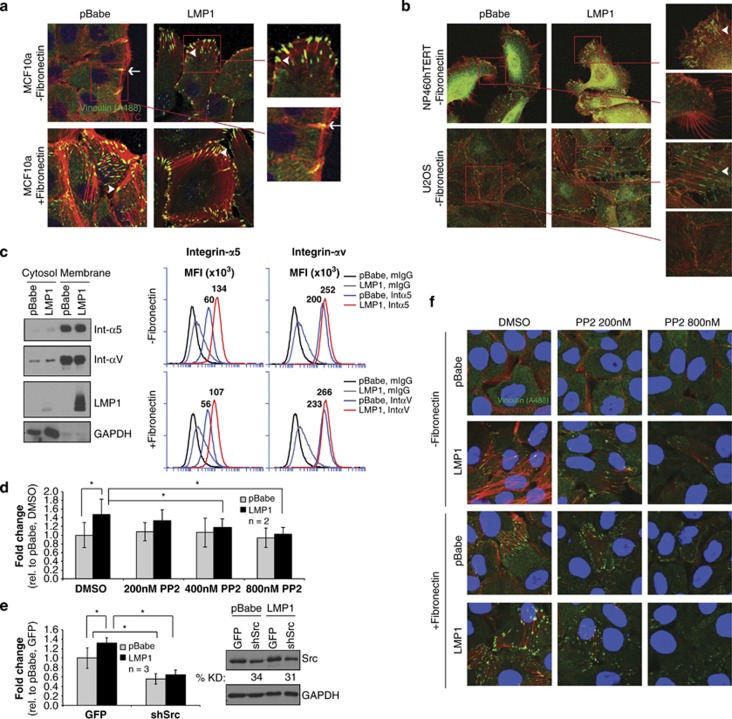
Src contributes to LMP1-induced FAs and migration to fibronectin. (**a**, **b**) Immunofluorescence staining of MCF10a, NP460hTERT and U2OS stable cell lines for FA markers (vinculin and phalloidin) and imaged by confocal microscopy. Enlarged images display individual FAs (arrowhead) and adherens junctions (arrow). (**c**) Immunoblot analysis for integrin-α5 and -αV from cytosolic and membrane fractions of MCF10a cell lines and flow cytometry analyses of non-permeabilized MCF10a cells for surface integrin-α5 and -αV. MFI, mean fluorescence intensity. (**d**, **e**) Inhibition of Src in MCF10a cells with (**d**) increasing doses of the PP2 Src kinase inhibitor and, (**e**) Src short hairpin RNA knockdown by transient transduction was analyzed by transwell migration to fibronectin. The average knockdown (% KD) from three independent experiments (*n*) is indicated, with one representative Src immunoblot shown. Fold-change in migration was calculated from independent experiments with error bars indicating s.d., and compared by the Student's *t*-test. *P=0.01. (**f**) Immunofluorescence staining for the assembly of FAs (vinculin and phalloidin) in MCF10a cells treated with PP2 at 200 nM and 800 nM, on fibronectin-coated and -uncoated surfaces.

Integrin binding to ECM initiates assembly of FAs leading to actin polymerization, migration, and the formation of filopodia and lamellipodia.^31^ To investigate potential LMP1-mediated effects, FAs were imaged by vinculin staining and by detection of polymerized actin with phalloidin (Figure 5a and b). In MCF10a cells on uncoated surfaces, vinculin was predominantly cytoplasmic with few FAs in pBabe cells and specific foci colocalized at intercellular regions indicative of adherens junctions (Figure 5a, arrow). In contrast, abundant FAs, identified by punctate vinculin staining and emanating strands of polymerized actin, were detected in LMP1-expressing cells indicating that LMP1 efficiently induced the assembly of FAs without exogenous addition of fibronectin coating (Figure 5a, arrowhead). FAs detected in LMP1-expressing cells were more abundant along the cell periphery, illustrated by lamellipodia structures lining the leading edge. On fibronectin-coated surfaces, both pBabe and LMP1-expressing MCF10a cells induced abundant FAs with prominent filopodia and lamellipodia (Figure 5a). The induction of FAs in the absence of fibronectin coating was further evaluated in additional epithelial cell lines, NP460hTERT and U2OS (Figure 5b). In hTERT-immortalized NP460 nasopharyngeal cells, stable expression of LMP1-induced FAs similar to the effect in MCF10a cells (Figure 5b). Basal levels of FAs are higher in U2OS cells and predominantly localize to the cell periphery; however, LMP1 further increased the number of FAs localized throughout the cell (Figure 5b). These data support that LMP1 promotes assembly of FAs in multiple epithelial cell types. In addition, the relative levels of LMP1 expression varied between stable cell lines but were comparable to the range detected in EBV-infected epithelial and B cells, and an NPC tumor (Supplementary Figure 1). By targeting a conserved epitope and nucleotide sequence in LMP1 strain variants, the relative LMP1 levels were higher in the NPC xenograft (C15 tumor) compared with trace levels in cultured epithelial cell lines (293, NP460hTERT, HK1) infected *de novo* (Supplementary Figure 1A), indicating that even trace levels of LMP1 in stable NP460hTERT cells, comparable to the low levels in EBV-infected cultured epithelial cells, was sufficient to activate the induction of FAs.

To further investigate if LMP1 promotes the assembly of FAs by modulating total integrin levels, MCF10a cells were analyzed by fractionation and flow cytometry for surface integrins. Detection of transmembrane LMP1, but not soluble GAPDH, indicated enrichment of membrane proteins. Integrin-α5 and -αV levels were equivalent in pBabe and LMP1-expressing cells, supporting that LMP1 does not promote FA assembly by regulating total integrin levels (Figure 5c). In contrast, flow cytometry detected increased cell surface levels of integrin-α5, but not integrin-αV, by LMP1 expression (Figure 5c). However, surface levels of integrin-α5 were not affected by fibronectin coating suggesting that additional factors may contribute to induction of FAs.

FAK and Src are recruited to FA signaling complexes to mediate downstream intracellular signaling. Src inhibition prevents the activation of Rac, Rho and Cdc42, impeding actin polymerization and migration.^31^ To investigate the role of FA signaling in LMP1-induced migration, Src was inhibited with the Src family kinase inhibitor PP2 or by short hairpin RNA knockdown in MCF10a cells, and assessed by transwell migration to fibronectin. Treatment of MCF10a cells with PP2 at 400 and 800 nM decreased LMP1-induced migration (Figures 5d, *P*=0.01), but did not affect control cell migration. Transient knockdown of Src further decreased migration in LMP1-expressing cells, which also occurred in pBabe cells and may be indicative of a kinase-independent effect not specific to LMP1 expression (Figures 5e, *P*=0.01). To assess the effects of Src inhibition on FA assembly, MCF10a cells were treated with two doses of PP2 (200 and 800 nM). At the sub-optimal inhibitory dose (200 nM), residual levels of actin polymerization were still evident but were inhibited by the higher PP2 dose (800 nM; Figure 5f). The induction of actin polymerization by LMP1 in the absence of fibronectin-coating was also inhibited by PP2 at 800 nM (Figure 5f). These findings reveal that LMP1 promotes formation of FAs, and downstream activation of Src is required for LMP1-induced migration.

### LMP1 promotes the recruitment of integrin-α5 and Src to FAs

**Figure 6:**
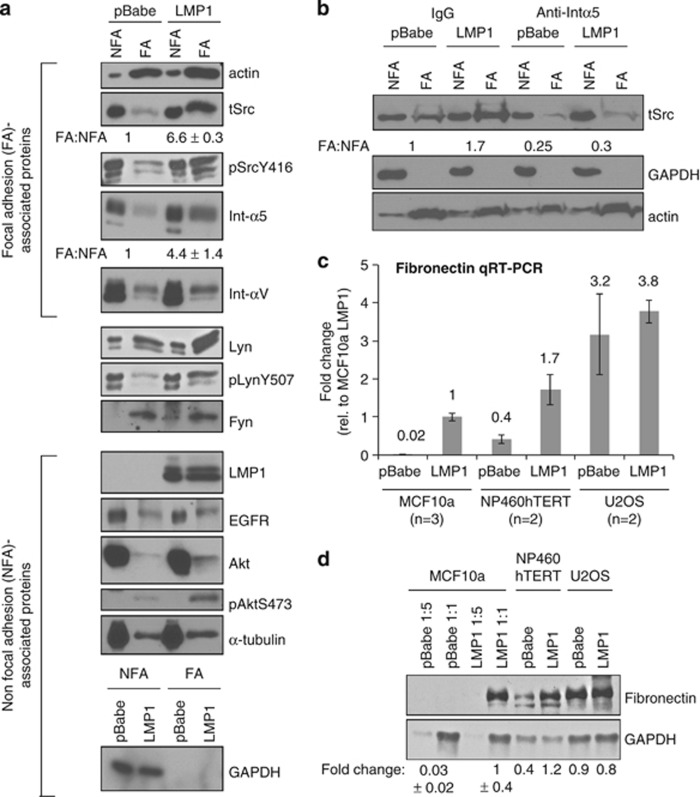
LMP1 promotes surface recruitment of integrin-α5 and production of fibronectin. (**a**) Immunoblot analysis of NFA and FA-enriched fractions for recruitment of FA-associated proteins in MCF10a stable cell lines. The fold-change of Src and integrin-α5 levels from two independent experiments, relative to pBabe cells (set to 1) is represented as a ratio of FA:NFA levels. (**b**) The effect of neutralizing antibodies to integrin-α5 on the recruitment of Src to FAs was analyzed by immunoblotting. (**c**, **d**) Quantitative RT–PCR and immunoblot analysis of fibronectin in MCF10a, NP460hTERT and U2OS cells. (**c**) Fibronectin transcript was normalized to GAPDH with fold-change quantified from biological replicas (*n*), relative to MCF10a LMP1 cells set to 1. (**d**) Fibronectin expression was detected by immunoblot and quantified on LICOR Odyssey Infrared Imager. Fold-change in MCF10a cells was determined from triplicate biological replicas.

To determine the potential mechanism for LMP1-induced FAs, FA complexes were isolated using fibronectin-coated magnetic beads and analyzed for associated proteins. The fibronectin-bound complexes were lysed in non-ionic detergent, and both non-FA (NFA) and FA-enriched fractions were collected and assessed for FA-enriched proteins (actin, Src, integrin-α5 and -αV) or NFA proteins, including plasma membrane-associated [epidermal growth factor receptor (EGFR) and protein kinase B (Akt)] and cytosolic (GAPDH) proteins. In the FA fraction, detection of low levels of epidermal growth factor receptor (EGFR) and protein kinase B (Akt) without GAPDH indicated co-purification with trace levels of integral and plasma membrane-associated proteins, but may also suggest cross-talk between integrin, epidermal growth factor receptor (EGFR) and protein kinase B (Akt) pathways (Figure 6a).^32, 33^ In contrast, high levels of actin were detected in the FA fraction. Phosphorylated Src (Y416) and Lyn (Y507) in the FA fraction were selectively increased in LMP1-expressing cells, and although these tyrosine-specific antibodies may cross-react with other Src family kinase members, total levels of Src and Lyn were also correspondingly increased (Figure 6a). Quantitation of recruitment to the FA fraction (FA:NFA ratio) indicated selective enrichment of Src and integrin-α5 in LMP1-expressing cells (6.6-fold and 4.4-fold, respectively) without affecting integrin-αV levels (Figure 6a). LMP1 was detected in both NFA and FA fractions, suggesting that LMP1 may associate with FA complexes (Figure 6a). To further analyze if the LMP1-mediated selective enrichment of Src to FAs is dependent on integrin-α5, MCF10a cells were pretreated with integrin-α5 neutralizing antibodies and re-analyzed for FA complexes. Pretreatment with integrin-α5 neutralizing antibodies inhibited Src recruitment to the FA fraction, which was modestly increased in LMP1-expressing cells treated with isotype control antibody (1.7-fold; Figure 6b). In addition, LMP1 may also induce FAs by increasing production of fibronectin. LMP1 expression in MCF10a and NP460hTERT cells increased fibronectin transcripts and protein levels (Figure 6c and d); however, this was only detectable in cell lines with low (MCF10a) or intermediate (NP460hTERT) levels of basal fibronectin, and not in cells with high basal levels (U2OS). Collectively, these findings indicate that LMP1-mediated induction of fibronectin, integrin-α5-dependent FAs and N-cadherin ligations represents multiple mechanisms by which LMP1 targets extracellular interactions to promote adhesive and migratory properties in epithelial cells.

## Discussion

Expression of LMP1 in rodent fibroblast and epithelial carcinoma cell lines promotes cell growth and motility.^4, 6^ LMP1 regulates cell motility by the modulation of junctional proteins including plakoglobin and E-cadherin, secretion of the matrix metalloproteinase 9 and potentially through the induction of N-cadherin.^7, 8, 10, 11, 14, 19, 34, 35^ In this study, LMP1-mediated effects on cell surface interactions were further evaluated for integrin and cadherin-mediated interactions in the pre-malignant cell line MCF10a. The findings indicate that LMP1 promotes the spontaneous assembly of FAs, potentially through increased production of ECM proteins, and increases the adhesion and transwell motility to fibronectin by promoting integrin-α5-mediated FAs.^36^ The inhibition of migration by N-cadherin neutralizing antibody indicates that intercellular N-cadherin ligation may promote collective cell migration or inhibit downstream pro-migratory signaling mechanisms. For instance, blocking N-cadherin ligation can prevent its association with the receptor tyrosine kinase fibroblast growth factor receptor (FGFR), resulting in the inhibition of extracellular signal-regulated kinase (ERK)-dependent activation of matrix metalloproteinase 9 transcription, thus inhibiting invasion of breast cancer epithelial cells.^3, 37^ The potential of N-cadherin as a therapeutic target has also been explored in xenograft models of melanoma, prostate and pancreatic cancers by inhibiting N-cadherin ligation with peptide antagonists and blocking antibodies.^38, 39, 40^

The process of epithelial cell adhesion to the substratum, and suspended migration to ECM proteins, is a dynamic process that involves opposing interactions with the stroma. ECM proteins such as fibronectin exist as a substratum-bound form and soluble ligand in plasma that are mimicked by adhesion and transwell migration assays, respectively. Migration of cells to these forms is defined as haptotactic and chemotactic migration, respectively, and may be regulated by differential signaling mechanisms.^41^ Metastatic cells must acquire the ability to detach from the primary site, sequentially intra- and extra-vasate from endothelial cells, and finally re-attach at secondary sites.^18^ These reciprocal changes are also observed at the molecular level by reversal of EMT to MET (mesenchymal–epithelial transition) as cells migrate to distal sites.^18^ Therefore, the ability of LMP1 to increase adhesion and chemotaxis to fibronectin may mimic these cycling processes during metastasis.

In response to fibronectin, LMP1 specifically recruited integrin-α5, but not integrin-αV, in mediating adhesion and motility (Figures 3 and 4). The induction of integrin-mediated effects by LMP1 was not mediated by changes in total integrin-α5 levels, and although surface integrin-α5 levels were unaffected by fibronectin coating, LMP1 may promote conformationally active integrins once recruited to the cell surface (Figure 5c). In addition, the increased deposition of fibronectin by LMP1 may directly contribute to the activation of FAs (Figure 6c and d). Previous studies in NPC cell lines and biopsies have identified additional LMP1-induced integrins including integrin-α6 and -αV.^42, 43^ Interestingly, integrin-α6 expression correlates with LMP2A levels and contributed to LMP2A-mediated migration and invasion of primary epithelial cells.^44^ In addition, LMP2A greatly increases membrane-localized integrin-αV and blocking integrin-αV ligation prevents LMP2A-induced migration to fibronectin, collagen and serum.^30^ The respective preference of LMP1 and LMP2A for integrin-α5 and -αV could reflect the selection of integrin-α5 for fibronectin and the broader ligand repertoire of integrin-αV, and may allow LMP1 and LMP2A to respond to changing extracellular environments.

Integrins are also required for EBV infection of epithelial cells and localize to the virological synapse, which mediates transfer of cell-associated virions to epithelial cells that do not express the primary EBV receptor, CD21.^45^ Transfer of cell-associated virus and direct cell-free binding can be blocked by competition with peptides or natural integrin ligands, such as fibronectin and vitronectin, or by specific knockdown of integrin-αV to inhibit fusion of the EBV gHgL 2-part complex with epithelial cells.^45, 46^ Therefore, integrins participate in multiple mechanisms of EBV pathogenesis in epithelial cells including LMP1- and LMP2A-induced cell migration and EBV infection.

Interaction of tumor cells with the microenvironment is thought to also contribute to initiation of metastasis. It is postulated that the switch to metastatic outgrowth is mediated by ECM proteins, integrin activation and actin stress fiber remodeling.^47, 48^ Fibronectin is associated with poor prognosis in multiple types of epithelial cell malignancies and is also induced in NPC in association with EMT markers.^49, 50^ In this study, the enhanced response of LMP1-expressing cells to fibronectin and the induction of EMT could potentially indicate a physiologically relevant role for fibronectin in the metastatic phenotype of NPC. In summary, this study indicates that LMP1 can modulate cell surface interactions in addition to previously defined intracellular signaling pathways, to regulate adhesive and motile phenotypes. The induction of FAs and the ligation of N-cadherin suggest that LMP1 modulates the individual and collective migration of epithelial cells. These mechanisms mediated by LMP1, and also possibly by LMP2A, likely contribute to NPC metastasis, and it is possible that the targeting of N-cadherin and integrin signaling may have therapeutic potential for the treatment of metastatic NPC.

## Materials and Methods

### Cell lines

MCF10a, NP460hTERT, C666-1 cells were maintained as previously described and U2OS cells were maintained in Dulbecco's modified Eagle's medium with 10% fetal bovine serum.^14, 25, 51, 52^ C666-1 cells are derived from an EBV-positive NPC tumor, but express negligible amounts of endogenous LMP1.^8, 14, 53^ Stable cell lines were generated using pBabe retrovirus expressing the LMP1 B958 strain.^7^ For fibronectin coating, 50 μg/ml fibronectin (Sigma, St Louis, MO, USA) was applied to tissue culture plates overnight at 4 ^o^C and rinsed on day of use.

### Quantitative RT–PCR

Total RNA was purified with the RNeasy Mini Kit and 50 ng of RNA was analyzed by the Quantifast Sybr-green RT-PCR kit (Qiagen, Venlo, Netherlands). Primers were as follows, N-cadherin (5′-AAATTGAGCCTGAAGCCAAC-3′ and 5′-GTGGCCACTGTGCTTACTGA-3′); E-cadherin (5′-CCTGGGACTCCACCTACAGA-3′ and 5′-CTGCTTGGATTCCAGAAACG-3′); and GAPDH (5′-TGCACCACCAACTGCTTAGC-3′ and 5′-GAGGGGCCATCCACAGTCTT-3′). For fibronectin and LMP1 quantitation: fibronectin (5′-ACTGAGACTCCGAGTCAGCC-3′ and 5′-TTCCAACGGCCTACAGAATT-3′); LMP1 (5′-ACCTTCTCTGTCCACTTGGA-3′ and 5′-AGAGTCCACCAGTTTTGTTG-3′) and human-specific GAPDH to avoid detection of mouse GAPDH in NPC xenografts, HsGAPDH (5′-AATCAAGTGGGGCGATGCTG-3′ and 5′-GTTCACACCCATGACGAACATG-3′). Reactions were performed on ABI 7900HT and StepOne Plus instruments (Applied Biosystems, Waltham, MA, USA), values were normalized to GAPDH and relative fold-change was calculated by the ΔΔCt comparative method.

### Immunoblot and immunofluorescence

Whole cell, cytosolic and membrane lysates were prepared as previously described.^7, 30^ Cell lysates were resolved on sodium dodecyl sulfate–polyacrylamide gel electrophoresis, immunoblotted and quantified by Image J densitometry (NIH, Bethesda, MD, USA) or on the LICOR Odyssey Imager (Lincoln, NE, USA). For immunofluorescence, cells were fixed in 4% paraformaldehyde, permeabilized with 0.1% Triton X-100 and stained for FAs with vinculin antibody (1:400, Sigma) and TRITC-Phalloidin (0.2 μg/ml, Sigma) according to the Focal Adhesion Staining Kit (Millipore, Billerica, MA, USA). Immunofluorescence of N-cadherin, E-cadherin and β-catenin was performed with the following modifications: 5% bovine serum albumin block and incubation with 2.5 μg/ml primary antibody in block overnight at 4 ^o^C. Fluorescent secondary antibodies were used at 5–10 μg/ml and counterstained with 4,6-diamidino-2-phenylindole or Draq5. Antibodies were from: BD Biosciences (San Jose, CA, USA) (E-cadherin, N-cadherin, β-catenin, integrin-α5, integrin-αV); Santa Cruz Biotechnology (Dallas, TX, USA) (GAPDH, HSC70, β-catenin, Lyn, Fyn, α−tubulin); Sigma (actin, vinculin); Cell Signaling Technologies (Danvers, MA, USA) (Akt, pAkt, Src, pSrc, pLyn); Amersham (Marlborough, MA, USA) (horseradish peroxidase-conjugated anti-mouse or anti-rabbit) and Invitrogen (Grand Island, NY, USA) (Alexa Fluor 488 anti-mouse IgG and Alexa Fluor 647 anti-rabbit IgG). EGFR antibody was a gift from Dr Shelton Earp (UNC-Chapel Hill, NC, USA). LMP1 antibodies were from Santa Cruz Biotechnology (clones CS1-4); Ascenion (Munich, Germany) (clones 8G3, 7G8, 7E10) or was produced from the S12 hybridoma.^54^ Cells were imaged with confocal microscopes [Zeiss 710 (Jena, Germany) and Olympus FV500 (Center Valley, PA, USA), University of North Carolina Microscopy Services Laboratory; and Leica TCS SP2 upright (Wetzlar, Germany), University of Pittsburgh Center for Biologic Imaging], acquired on a 63X oil objective, with brightness and contrast equally adjusted across images.

### Adhesion and transwell migration assays

Adhesion was measured with the xCelligence RTCA DP instrument (ACEA Biosciences, San Diego, CA, USA). Trypsin-EDTA dissociated cells (20 000) were seeded in 16-well electronic plates (E-plate 16), with or without fibronectin coating (50 μg/ml). Blocking antibodies at 20 μg/ml (anti-integrin α5 clone P1D6, Millipore; anti-integrin αV clone NKI-M9, Biolegend (San Diego, CA, USA); or mouse IgG, Sigma) was incubated with dissociated cells for 30 min at 4 ^o^C to minimize antibody internalization before seeding. Electrical impedance was measured for a minimum of triplicate wells and blanked to wells with media alone. Transwell migration was performed as previously described with the following modifications:^8^ dissociation in 0.05% trypsin-EDTA for cadherin cleaving conditions, or 0.1% trypsin in 2 mM CaCl_2_ and passed through a cell strainer to obtain single cells for cadherin preserving conditions.^28^ The underside of transwell was pre-coated with 10 μg/ml fibronectin and 100 000 cells were seeded in reduced assay media (1% horse serum, no EGF). Blocking treatments were performed by pre-incubation with neutralizing integrin antibodies (20 μg/ml for integrin-α5 and 50 μg/ml for integrin-αV), or equivalent amounts of mouse IgG control for 30 min at 4 ^o^C before seeding in transwells. Knockdown of Src was performed by transient transduction with Src short hairpin RNA retrovirus expressed from pRetroSuper (Addgene #26982, Cambridge, MA, USA). Each biological replica experiment (*n*) was assayed in duplicate wells and imaged for quadruplicate fields per well. Migrated cells were counted using Image J software.

### Flow cytometry

Live cells were stained for surface integrins with 1 μg primary antibody (anti-integrin-α5 clone P1D6, anti-integrin-αV clone NKI-M9 or isotype control) and 1 μg secondary antibody (Alexa Fluor 488 anti-mouse IgG). Incubations were performed in 1% fetal bovine serum stain buffer for 20 min at 4 ^o^C, and analyzed on the BD Accuri C6 cytometer (BD Biosciences) (UPCI Cytometry Facility).

### FA enrichment

Fibronectin-coated magnetic beads (1 X 10^9^ Epoxy M-270 beads, Invitrogen) were blocked in 5% bovine serum albumin for 1 h at 37 ^o^C, and resuspended to ~1 million beads/ml in phosphate-buffered saline. Sonicated beads (40 μl) were added to adherent cells pre-starved for 2 h in 0.5% delipidated bovine serum albumin (to minimize Rho activation). For antibody blocking, cells were pre-incubated with 10 μg/ml anti-integrin α5 antibody for 30 min at 4 ^o^C. At 2 h, unbound beads were removed in cold TBS-M (Tris-buffered saline with 1 mM MgCl_2_ to preserve intact FAs) and cells with mature FAs bound to beads were lysed (20 mM Tris pH 7.6, 150 mM NaCl, 0.1% NP-40, 2 mM MgCl_2_; supplemented with protease and phosphatase inhibitor cocktails, Sigma). The FA fraction was isolated by magnetic enrichment and eluted by boiling in 2X sodium dodecyl sulfate–polyacrylamide gel electrophoresis sample buffer. The unbound fraction was collected as the NFA fraction. Eluted proteins were analyzed by immunoblotting using equal volumes of the same fraction equivalent to 1:200 NFA:FA cell equivalents.

### Calcium switch assay

Cells were washed in calcium and magnesium-free phosphate-buffered saline and treated with 4 mM EDTA for 30 min at 37 ^o^C to dissociate ligated cadherins. To initiate a calcium switch, MCF10a complete growth medium was replenished and lysates from triton-solubility assays were prepared at indicated time points as previously described.^14^ Equal number of cell equivalents (1:1) from triton-soluble and triton-insoluble fractions were analyzed by immunoblotting.

### Statistics

All statistics was performed by two-tailed Student's *t*-test, with biological and technical replicates displayed in each figure legend. *P*-values were calculated using the most stringent paired *t*-test method. For comparisons of dissimilar variances calculated by F-test, s.d. are displayed for each individual experiment over multiple time points (Figure 4).

## References

[bib1] 1Cohen JI, Fauci AS, Varmus H, Nabel GJ. Epstein-Barr virus: an important vaccine target for cancer prevention. Sci Transl Med 2011; 3: 107fs7. 10.1126/scitranslmed.3002878 PMC3501269 22049067

[bib2] 2Raab-Traub N. Pathogenesis of Epstein-Barr virus and its associated malignancies. Semin Virol 1996; 7: 315–323.

[bib3] 3Temple RM, Zhu J, Budgeon L, Christensen ND, Meyers C, Sample CE. Efficient replication of Epstein-Barr virus in stratified epithelium *in vitro*. Proc Natl Acad Sci USA 2014; 111: 16544–16549. 25313069 10.1073/pnas.1400818111 PMC4246336

[bib4] 4Raab-Traub N. Novel mechanisms of EBV-induced oncogenesis. Curr Opin Virol 2012; 2: 453–458. 22858118 10.1016/j.coviro.2012.07.001 PMC4617531

[bib5] 5Raab-Traub N. Epstein-Barr virus in the pathogenesis of NPC. Semin Cancer Biol 2002; 12: 431–441. 12450729 10.1016/s1044579x0200086x

[bib6] 6Dawson CW, Port RJ, Young LS. The role of the EBV-encoded latent membrane proteins LMP1 and LMP2 in the pathogenesis of nasopharyngeal carcinoma (NPC). Semin Cancer Biol 2012; 22: 144–153. 22249143 10.1016/j.semcancer.2012.01.004

[bib7] 7Mainou BA, Everly DN Jr., Raab-Traub N. Epstein-Barr virus latent membrane protein 1 CTAR1 mediates rodent and human fibroblast transformation through activation of PI3K. Oncogene 2005; 24: 6917–6924. 16007144 10.1038/sj.onc.1208846

[bib8] 8Shair KH, Schnegg CI, Raab-Traub N. EBV latent membrane protein 1 effects on plakoglobin, cell growth, and migration. Cancer Res 2008; 68: 6997–7005. 18757414 10.1158/0008-5472.CAN-08-1178 PMC2593097

[bib9] 9Shair KH, Bendt KM, Edwards RH, Nielsen JN, Moore DT, Raab-Traub N. Epstein-Barr virus-encoded latent membrane protein 1 (LMP1) and LMP2 A function cooperatively to promote carcinoma development in a mouse carcinogenesis model. J Virol 2012; 86: 5352–5365. 22357283 10.1128/JVI.07035-11 PMC3347346

[bib10] 10Dawson CW, Laverick L, Morris MA, Tramoutanis G, Young LS. Epstein-Barr virus-encoded LMP1 regulates epithelial cell motility and invasion via the ERK-MAPK pathway. J Virol 2008; 82: 3654–3664. 18199641 10.1128/JVI.01888-07 PMC2268486

[bib11] 11Kung CP, Meckes DG Jr., Raab-Traub N. Epstein-Barr virus LMP1 activates EGFR, STAT3, and ERK through effects on PKCdelta. J Virol 2011; 85: 4399–4408. 21307189 10.1128/JVI.01703-10 PMC3126279

[bib12] 12Mosialos G, Birkenbach M, Yalamanchili R, VanArsdale T, Ware C, Kieff E. The Epstein-Barr virus transforming protein LMP1 engages signaling proteins for the tumor necrosis factor receptor family. Cell 1995; 80: 389–399. 7859281 10.1016/0092-8674(95)90489-1

[bib13] 13Horikawa T, Yang J, Kondo S, Yoshizaki T, Joab I, Furukawa M et al. Twist and epithelial-mesenchymal transition are induced by the EBV oncoprotein latent membrane protein 1 and are associated with metastatic nasopharyngeal carcinoma. Cancer Res 2007; 67: 1970–1978. 17332324 10.1158/0008-5472.CAN-06-3933

[bib14] 14Shair KH, Schnegg CI, Raab-Traub N. Epstein-Barr virus latent membrane protein-1 effects on junctional plakoglobin and induction of a cadherin switch. Cancer Res 2009; 69: 5734–5742. 19584275 10.1158/0008-5472.CAN-09-0468 PMC2771661

[bib15] 15Tsai CN, Tsai CL, Tse KP, Chang HY, Chang YS. The Epstein-Barr virus oncogene product, latent membrane protein 1, induces the downregulation of E-cadherin gene expression via activation of DNA methyltransferases. Proc Natl Acad Sci USA 2002; 99: 10084–10089. 12110730 10.1073/pnas.152059399 PMC126628

[bib16] 16Luo WR, Wu AB, Fang WY, Li SY, Yao KT. Nuclear expression of N-cadherin correlates with poor prognosis of nasopharyngeal carcinoma. Histopathology 2012; 61: 237–246. 22385354 10.1111/j.1365-2559.2012.04212.x

[bib17] 17Tsao SW, Liu Y, Wang X, Yuen PW, Leung SY, Yuen ST et al. The association of E-cadherin expression and the methylation status of the E-cadherin gene in nasopharyngeal carcinoma cells. Eur J Cancer 2003; 39: 524–531. 12751385 10.1016/s0959-8049(02)00494-x

[bib18] 18Kalluri R, Weinberg RA. The basics of epithelial-mesenchymal transition. J Clin Invest 2009; 119: 1420–1428. 19487818 10.1172/JCI39104 PMC2689101

[bib19] 19Kim KR, Yoshizaki T, Miyamori H, Hasegawa K, Horikawa T, Furukawa M et al. Transformation of Madin-Darby canine kidney (MDCK) epithelial cells by Epstein-Barr virus latent membrane protein 1 (LMP1) induces expression of Ets1 and invasive growth. Oncogene 2000; 19: 1764–1771. 10777210 10.1038/sj.onc.1203502

[bib20] 20Lo AK, Liu Y, Wang XH, Huang DP, Yuen PW, Wong YC et al. Alterations of biologic properties and gene expression in nasopharyngeal epithelial cells by the Epstein-Barr virus-encoded latent membrane protein 1. Lab Invest 2003; 83: 697–709. 12746479 10.1097/01.lab.0000067480.44925.10

[bib21] 21Hazan RB, Qiao R, Keren R, Badano I, Suyama K. Cadherin switch in tumor progression. Ann N Y Acad Sci 2004; 1014: 155–163. 15153430 10.1196/annals.1294.016

[bib22] 22Dawson CW, Tramountanis G, Eliopoulos AG, Young LS. Epstein-Barr virus latent membrane protein 1 (LMP1) activates the phosphatidylinositol 3-kinase/Akt pathway to promote cell survival and induce actin filament remodeling. J Biol Chem 2003; 278: 3694–3704. 12446712 10.1074/jbc.M209840200

[bib23] 23Liu HP, Chen CC, Wu CC, Huang YC, Liu SC, Liang Y et al. Epstein-Barr virus-encoded LMP1 interacts with FGD4 to activate Cdc42 and thereby promote migration of nasopharyngeal carcinoma cells. PLoS Pathog 2012; 8: e1002690. 22589722 10.1371/journal.ppat.1002690 PMC3349753

[bib24] 24Puls A, Eliopoulos AG, Nobes CD, Bridges T, Young LS, Hall A. Activation of the small GTPase Cdc42 by the inflammatory cytokines TNF(alpha) and IL-1, and by the Epstein-Barr virus transforming protein LMP1. J Cell Sci 1999; 112: (Pt 17) 2983–2992. 10444392 10.1242/jcs.112.17.2983

[bib25] 25Chan SY, Choy KW, Tsao SW, Tao Q, Tang T, Chung GT et al. Authentication of nasopharyngeal carcinoma tumor lines. Int J Cancer 2008; 122: 2169–2171. 18196576 10.1002/ijc.23374

[bib26] 26Maeda M, Johnson KR, Wheelock MJ. Cadherin switching: essential for behavioral but not morphological changes during an epithelium-to-mesenchyme transition. J Cell Sci 2005; 118: 873–887. 15713751 10.1242/jcs.01634

[bib27] 27Marambaud P, Wen PH, Dutt A, Shioi J, Takashima A, Siman R et al. A CBP binding transcriptional repressor produced by the PS1/epsilon-cleavage of N-cadherin is inhibited by PS1 FAD mutations. Cell 2003; 114: 635–645. 13678586 10.1016/j.cell.2003.08.008

[bib28] 28von Schlippe M, Marshall JF, Perry P, Stone M, Zhu AJ, Hart IR. Functional interaction between E-cadherin and alphav-containing integrins in carcinoma cells. J Cell Science 2000; 113: 425–437. 10639330 10.1242/jcs.113.3.425

[bib29] 29Takeichi M. Functional correlation between cell adhesive properties and some cell surface proteins. J Cell Biol 1977; 75: 464–474. 264120 10.1083/jcb.75.2.464 PMC2109947

[bib30] 30Fotheringham JA, Coalson NE, Raab-Traub N. Epstein-Barr virus latent membrane protein-2A induces ITAM/Syk- and Akt-dependent epithelial migration through alphav-integrin membrane translocation. J Virol 2012; 86: 10308–10320. 22837212 10.1128/JVI.00853-12 PMC3457296

[bib31] 31Guo W, Giancotti FG. Integrin signalling during tumour progression. Nat Rev Mol Cell Biol 2004; 5: 816–826. 15459662 10.1038/nrm1490

[bib32] 32Bill HM, Knudsen B, Moores SL, Muthuswamy SK, Rao VR, Brugge JS et al. Epidermal growth factor receptor-dependent regulation of integrin-mediated signaling and cell cycle entry in epithelial cells. Mol Cell Biol 2004; 24: 8586–8599. 15367678 10.1128/MCB.24.19.8586-8599.2004 PMC516761

[bib33] 33Clark EA, King WG, Brugge JS, Symons M, Hynes RO. Integrin-mediated signals regulated by members of the rho family of GTPases. J Cell Biol 1998; 142: 573–586. 9679153 10.1083/jcb.142.2.573 PMC2133065

[bib34] 34Mainou BA, Everly DN Jr., Raab-Traub N. Unique signaling properties of CTAR1 in LMP1-mediated transformation. J Virol 2007; 81: 9680–9692. 17626074 10.1128/JVI.01001-07 PMC2045399

[bib35] 35Takeshita H, Yoshizaki T, Miller WE, Sato H, Furukawa M, Pagano JS et al. Matrix metalloproteinase 9 expression is induced by Epstein-Barr virus latent membrane protein 1 C-terminal activation regions 1 and 2. J Virol 1999; 73: 5548–5555. 10364303 10.1128/jvi.73.7.5548-5555.1999 PMC112612

[bib36] 36Ma LJ, Lee SW, Lin LC, Chen TJ, Chang IW, Hsu HP et al. Fibronectin overexpression is associated with latent membrane protein 1 expression and has independent prognostic value for nasopharyngeal carcinoma. Tumour Biol 2014; 35: 1703–1712. 24081675 10.1007/s13277-013-1235-8

[bib37] 37Suyama K, Shapiro I, Guttman M, Hazan RB. A signaling pathway leading to metastasis is controlled by N-cadherin and the FGF receptor. Cancer Cell 2002; 2: 301–314. 12398894 10.1016/s1535-6108(02)00150-2

[bib38] 38Augustine CK, Yoshimoto Y, Gupta M, Zipfel PA, Selim MA, Febbo P et al. Targeting N-cadherin enhances antitumor activity of cytotoxic therapies in melanoma treatment. Cancer Res 2008; 68: 3777–3784. 18483261 10.1158/0008-5472.CAN-07-5949

[bib39] 39Shintani Y, Fukumoto Y, Chaika N, Grandgenett PM, Hollingsworth MA, Wheelock MJ et al. ADH-1 suppresses N-cadherin-dependent pancreatic cancer progression. Int J Cancer 2008; 122: 71–77. 17721921 10.1002/ijc.23027

[bib40] 40Tanaka H, Kono E, Tran CP, Miyazaki H, Yamashiro J, Shimomura T et al. Monoclonal antibody targeting of N-cadherin inhibits prostate cancer growth, metastasis and castration resistance. Nat Med 2010; 16: 1414–1420. 21057494 10.1038/nm.2236 PMC3088104

[bib41] 41Aznavoorian S, Stracke ML, Parsons J, McClanahan J, Liotta LA. Integrin alphavbeta3 mediates chemotactic and haptotactic motility in human melanoma cells through different signaling pathways. J Biol Chem 1996; 271: 3247–3254. 8621727 10.1074/jbc.271.6.3247

[bib42] 42Lo AK, Liu Y, Wang X, Wong YC, Kai Fai Lee C, Huang DP et al. Identification of downstream target genes of latent membrane protein 1 in nasopharyngeal carcinoma cells by suppression subtractive hybridization. Biochim Biophys Acta 2001; 1520: 131–140. 11513954 10.1016/s0167-4781(01)00260-3

[bib43] 43Shi W, Bastianutto C, Li A, Perez-Ordonez B, Ng R, Chow KY et al. Multiple dysregulated pathways in nasopharyngeal carcinoma revealed by gene expression profiling. Int J Cancer 2006; 119: 2467–2475. 16858677 10.1002/ijc.22107

[bib44] 44Pegtel DM, Subramanian A, Sheen TS, Tsai CH, Golub TR, Thorley-Lawson DA. Epstein-Barr-virus-encoded LMP2A induces primary epithelial cell migration and invasion: possible role in nasopharyngeal carcinoma metastasis. J Virol 2005; 79: 15430–15442. 16306614 10.1128/JVI.79.24.15430-15442.2005 PMC1316046

[bib45] 45Shannon-Lowe CD, Neuhierl B, Baldwin G, Rickinson AB, Delecluse HJ. Resting B cells as a transfer vehicle for Epstein-Barr virus infection of epithelial cells. Proc Natl Acad Sci USA 2006; 103: 7065–7070. 16606841 10.1073/pnas.0510512103 PMC1459019

[bib46] 46Chesnokova LS, Nishimura SL, Hutt-Fletcher LM. Fusion of epithelial cells by Epstein-Barr virus proteins is triggered by binding of viral glycoproteins gHgL to integrins alphavbeta6 or alphavbeta8. Proc Natl Acad Sci USA 2009; 106: 20464–20469. 19920174 10.1073/pnas.0907508106 PMC2787161

[bib47] 47Barkan D, El Touny LH, Michalowski AM, Smith JA, Chu I, Davis AS et al. Metastatic growth from dormant cells induced by a col-I-enriched fibrotic environment. Cancer Res 2010; 70: 5706–5716. 20570886 10.1158/0008-5472.CAN-09-2356 PMC3436125

[bib48] 48Barkan D, Kleinman H, Simmons JL, Asmussen H, Kamaraju AK, Hoenorhoff MJ et al. Inhibition of metastatic outgrowth from single dormant tumor cells by targeting the cytoskeleton. Cancer Res 2008; 68: 6241–6250. 18676848 10.1158/0008-5472.CAN-07-6849 PMC2561279

[bib49] 49Barkan D, Green JE, Chambers AF. Extracellular matrix: a gatekeeper in the transition from dormancy to metastatic growth. Eur J Cancer 2010; 46: 1181–1188. 20304630 10.1016/j.ejca.2010.02.027 PMC2856784

[bib50] 50Kong QL, Hu LJ, Cao JY, Huang YJ, Xu LH, Liang Y et al. Epstein-Barr virus-encoded LMP2A induces an epithelial-mesenchymal transition and increases the number of side population stem-like cancer cells in nasopharyngeal carcinoma. PLoS Pathog 2010; 6: e1000940. 20532215 10.1371/journal.ppat.1000940 PMC2880580

[bib51] 51Debnath J, Muthuswamy SK, Brugge JS. Morphogenesis and oncogenesis of MCF-10 A mammary epithelial acini grown in three-dimensional basement membrane cultures. Methods 2003; 30: 256–268. 12798140 10.1016/s1046-2023(03)00032-x

[bib52] 52Tsang CM, Zhang G, Seto E, Takada K, Deng W, Yip YL et al. Epstein-Barr virus infection in immortalized nasopharyngeal epithelial cells: regulation of infection and phenotypic characterization. Int J Cancer 2010; 127: 1570–1583. 20091869 10.1002/ijc.25173

[bib53] 53Marquitz AR, Mathur A, Shair KH, Raab-Traub N. Infection of Epstein-Barr virus in a gastric carcinoma cell line induces anchorage independence and global changes in gene expression. Proc Natl Acad Sci USA 2012; 109: 9593–9598. 22647604 10.1073/pnas.1202910109 PMC3386136

[bib54] 54Mann KP, Staunton D, Thorley-Lawson DA. Epstein-Barr virus-encoded protein found in plasma membranes of transformed cells. J Virol 1985; 55: 710–720. 2991591 10.1128/jvi.55.3.710-720.1985 PMC255052

