# Supplementary material for: Epstein–Barr virus LMP1 induces focal adhesions and epithelial cell migration through effects on integrin-α5 and N-cadherin
Source: Oncogenesis. 2015 Oct 19;4(10):e171–. doi: 10.1038/oncsis.2015.31 (PMC4632092; doi:10.1038/oncsis.2015.31)
Supplement: Supplementary Figure Legend [file oncsis201531x1.doc]

Supplementary Figure 1. Immunoblot and qRT-PCR analysis of LMP1. (A, B) Immunoblot for LMP1 in C666-1, MCF10a, NP460hTERT and U2OS cells compared to EBV-infected (EBV+) NPC xenografts, epithelial and B cell lines. LMP1 was detected with rat (8G3, 7G8, 7E10) and mouse (CS1-4) monoclonal antibodies. The antibody cocktails detect both the LMP1 strain variant (Med+) in C15 xenograft and the B958 LMP1 reference strain expressed in the other cell lines, but the rat monoclonals recognize a conserved epitope in Med+ and B958 LMP1 strains. Asterisk (*) denotes full-length LMP1-specific band. (A) Quantitation of LMP1 transcript by qRT-PCR amplified with primers targeting a conserved sequence in all LMP1 strains.
