# Supplementary material for: Epstein–Barr virus LMP1 induces focal adhesions and epithelial cell migration through effects on integrin-α5 and N-cadherin
Source: Oncogenesis. 2015 Oct 19;4(10):e171–. doi: 10.1038/oncsis.2015.31 (PMC4632092; doi:10.1038/oncsis.2015.31)

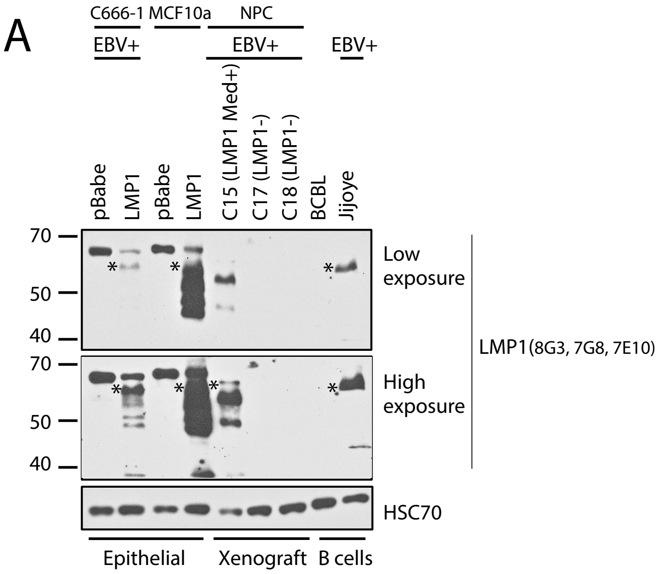

qRT-PCR for LMP1 transcript

Cell Line/ NPC Xenograft	LMP1 Transcript (% normalized to LCL)	Biological Replicates
LCL (EBV+)	100±2.9	3
BJAB	0	3
C15 (1) (EBV+/LMP1+)	116±6.1	2
C15 (2) (EBV+/LMP1+)	73.8±4.9	
C17 (1) (EBV+/LMP1-)	0	2
C17 (2) (EBV+/LMP1-)	0	
293 (EBV+)	6.5±0.98	3
293	0	3
NP460hTERT (EBV+)	0.8±0.013	3
NP460hTERT	0	3
HK1 (EBV+)	0.98±0.39	3
HK1	0	3

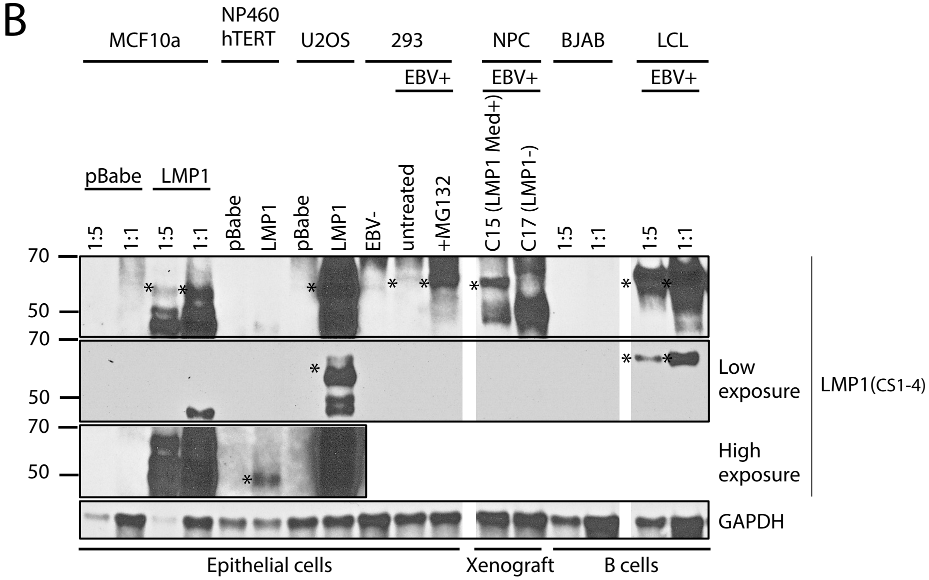

Supplement: Supplementary Figure 1 [file oncsis201531x2.pdf]
